# Imaging zinc trafficking *in vivo* by positron emission tomography with zinc-62

**DOI:** 10.1093/mtomcs/mfac076

**Published:** 2022-10-06

**Authors:** George Firth, Zilin Yu, Joanna J Bartnicka, David Parker, Jana Kim, Kavitha Sunassee, Hannah E Greenwood, Fahad Al-Salamee, Maite Jauregui-Osoro, Alberto Di Pietro, Joanna Guzman, Philip J Blower

**Affiliations:** School of Biomedical Engineering & Imaging Sciences, King's College London, St Thomas’ Hospital, London, SE1 7EH, UK; School of Biomedical Engineering & Imaging Sciences, King's College London, St Thomas’ Hospital, London, SE1 7EH, UK; School of Biomedical Engineering & Imaging Sciences, King's College London, St Thomas’ Hospital, London, SE1 7EH, UK; School of Physics and Astronomy, University of Birmingham, Edgbaston, Birmingham, B15 2TT, UK; School of Biomedical Engineering & Imaging Sciences, King's College London, St Thomas’ Hospital, London, SE1 7EH, UK; School of Biomedical Engineering & Imaging Sciences, King's College London, St Thomas’ Hospital, London, SE1 7EH, UK; School of Biomedical Engineering & Imaging Sciences, King's College London, St Thomas’ Hospital, London, SE1 7EH, UK; School of Biomedical Engineering & Imaging Sciences, King's College London, St Thomas’ Hospital, London, SE1 7EH, UK; School of Biomedical Engineering & Imaging Sciences, King's College London, St Thomas’ Hospital, London, SE1 7EH, UK; School of Biomedical Engineering & Imaging Sciences, King's College London, St Thomas’ Hospital, London, SE1 7EH, UK; School of Biomedical Engineering & Imaging Sciences, King's College London, St Thomas’ Hospital, London, SE1 7EH, UK; School of Biomedical Engineering & Imaging Sciences, King's College London, St Thomas’ Hospital, London, SE1 7EH, UK

**Keywords:** ^62^Zn, positron emission tomography, zinc trafficking, copper trafficking, PET metallomics, non-invasive imaging

## Abstract

Non-invasive imaging techniques to dynamically map whole-body trafficking of essential metals *in vivo* in health and diseases are needed. Despite ^62^Zn having appropriate physical properties for positron emission tomography (PET) imaging (half-life, 9.3 h; positron emission, 8.2%), its complex decay via ^62^Cu (half-life, 10 min; positron emission, 97%) has limited its use. We aimed to develop a method to extract ^62^Zn from a ^62^Zn/^62^Cu generator, and to investigate its use for *in vivo* imaging of zinc trafficking despite its complex decay. ^62^Zn prepared by proton irradiation of natural copper foil was used to construct a conventional ^62^Zn/^62^Cu generator. ^62^Zn was eluted using trisodium citrate and used for biological experiments, compared with ^64^Cu in similar buffer. PET/CT imaging and *ex vivo* tissue radioactivity measurements were performed following intravenous injection in healthy mice. [^62^Zn]Zn-citrate was readily eluted from the generator with citrate buffer. PET imaging with the eluate demonstrated biodistribution similar to previous observations with the shorter-lived ^63^Zn (half-life 38.5 min), with significant differences compared to [^64^Cu]Cu-citrate, notably in pancreas (>10-fold higher at 1 h post-injection). Between 4 and 24 h, ^62^Zn retention in liver, pancreas, and kidney declined over time, while brain uptake increased. Like ^64^Cu, ^62^Zn showed hepatobiliary excretion from liver to intestines, unaffected by fasting. Although it offers limited reliability of scanning before 1 h post-injection, ^62^Zn-PET allows investigation of zinc trafficking *in vivo* for >24 h and hence provides a useful new tool to investigate diseases where zinc homeostasis is disrupted in preclinical models and humans.

## Graphical Abstract

**Graphical Abstract:**
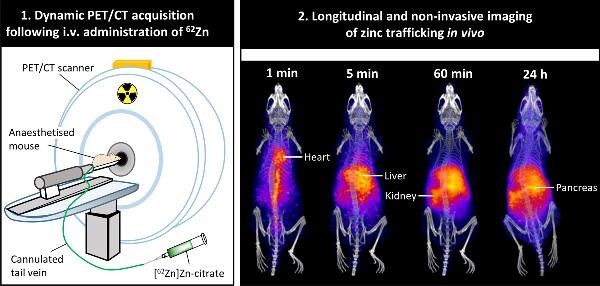
Imaging zinc trafficking *in vivo* with ^62^Zn-PET.

## Introduction

With an estimated 10% of the human genome encoding zinc proteins,^1^ zinc homeostasis is vital for normal physiological function. Dysregulation in zinc trafficking has been reported in several diseases of high socio-economic impact, including cancer,^2–6^ Alzheimer's disease,^7^^,^^8^ and diabetes.^9–11^ In order to investigate the role that zinc trafficking plays in disease, a technique is required that can monitor these dynamic pathways in a non-invasive manner throughout the whole body in animals and humans. Inductively coupled plasma mass spectrometry (ICP-MS) and zinc-sensing fluorescent probes are powerful tools for quantifying endogenous zinc, but are limited to *in vitro* and invasively obtained *ex vivo* samples.^12^ Recent efforts have been made to adapt zinc-sensing contrast agents for *in vivo* use as medical imaging contrast agents to study zinc *in vivo*.^13–16^ These are potentially useful agents to study labile zinc pools, but tools providing complementary information on the dynamic flux of zinc within the body are lacking. Positron emission tomography (PET) is a non-invasive medical imaging technique that provides dynamic functional information on biological molecular processes by mapping positrons originating from radioactive isotopes. With positron-emitting radioisotopes of essential metals becoming increasingly available, PET imaging is emerging as a means of studying dynamic acute (real-time) trafficking of essential metals at the whole-body level.^17^^,^^18^ The metal most studied to date by this approach is copper,^19–24^ but positron-emitting radioisotopes of zinc have been sought recently for this purpose.^25^^,^^26^

Three radioisotopes of zinc have applications in studying zinc biology (Table 1). ^65^Zn has the longest half-life (t_1/2_ = 243.8 days), and has been used to investigate zinc absorption and metabolism in plants,^27^ rodents, and humans.^28–30^ Although it emits gamma photons and low-abundance positrons, it is not suitable for whole body *in vivo* imaging owing to its long half-life and consequent problems of high radiation absorbed dose, and difficult radioactive waste management; *in vivo* measurements with ^65^Zn are limited to biopsy of selected tissues and subsequent gamma counting, which require much smaller amounts of radioactivity than imaging. The favourable emission properties of ^63^Zn (β^+^, 93%) and short half-life (38.5 min) make it an excellent option for imaging short-term (< 2 h) zinc biodistribution.^25^^,^^26^ To study zinc trafficking for longer periods, a longer half-life positron emitter is needed.

**Table 1.** Positron-emitting radioisotopes of zinc that have potential applications as tools for metallomics investigations

Radio nuclide	Half-life (t_1/2_)	Decay mode^a^	Mean positron energy (MeV)	Daughter nuclide	Production method
^62^Zn	9.3 h	β^+^(8.2%), EC	0.26	^62^Cu^b^	Cyclotron: solid Cu foil target, ^63^Cu(p,2n)^62^Zn
^63^Zn	38.5 min	β^+^(93%), EC	0.99	^63^Cu	Cyclotron: liquid ^63^Cu nitrate target, ^63^Cu(p,n)^63^Zn
^65^Zn	243.8 d	β^+^(98%), EC	0.14	^65^Cu	Cyclotron: solid ^65^Cu target, ^65^Cu(p,n)^65^Zn

EC denotes electron capture. ^a^ Parentheses denote percentage intensity (*I*). ^b 62^Cu decays via β^+^ decay (t_1/2_ = 10 min, *I*β^+^ = 98%, Eβ^+^ = 2.91 MeV).

^62^Zn, previously used as the parent radionuclide in ^62^Zn/^62^Cu generators as the basis of ^62^Cu-radiopharmaceutical production,^31–34^ has a suitable half-life (9.3 h) but due to its complex decay mode it has not seen significant use in PET imaging. Only 8.2% of ^62^Zn decays yield a positron. However, each decay of ^62^Zn yields the high-abundance positron emitter ^62^Cu (t_1/2_ = 9.7 min, β^+^, 98%). Thus, in an imaging experiment with ^62^Zn, after radioactive equilibrium is reached (about 1 h) more than 90% of the emitted positrons are from the daughter radionuclide ^62^Cu, raising the question of whether images reflect ^62^Zn distribution or redistribution following decay to ^62^Cu. An important distinction is that the half-life of ^62^Zn is greater than that of ^62^Cu by a factor of 60. Consequently, if an equilibrium mixture of ^62^Zn and ^62^Cu is injected into a subject, 1 h later six half-lives of ^62^Cu will have elapsed and essentially all the injected ^62^Cu will have decayed. Thereafter, on average, almost all the positrons detected will have been emitted by a radionuclide (^62^Cu or ^62^Zn) that has spent more than 98% of its existence since the time of injection as zinc rather than copper. Therefore, it is highly likely that the location of the decay event will represent the result of trafficking of zinc rather than copper and that in most biological settings, the short half-life of ^62^Cu will not allow sufficient time for significant redistribution following the *in vivo* conversion to ^62^Cu. We therefore hypothesized that, despite the perceived challenges, ^62^Zn will be a useful radionuclide with which to image zinc trafficking *in vivo*. Hence, we decided to re-evaluate PET imaging with ^62^Zn as an ‘*in vivo* generator’ of ^62^Cu, by direct comparison of ^62^Zn biodistribution with that of the copper radioisotope ^64^Cu.

Here we describe a new low-cost manually operated ^62^Zn/^62^Cu radionuclide generator system based on a published design,^33^ and its subsequent use to produce ^62^Zn in a form suitable for *in vivo* PET imaging, aiming to determine whether this underutilised radionuclide can provide insight into the dynamics of zinc trafficking in models of health and disease over a time period that cannot be achieved with the shorter half-life radioisotope, ^63^Zn. We report for the first time the biodistribution of zinc administered intravenously (i.v.) as zinc citrate over a period of 24 h, where previously only 2 h was possible because of the short half-life of ^63^Zn. We also investigated the effect of fasting on hepatobiliary clearance of ^62^Zn.

## Methods

### General

Reagents and materials were purchased from Sigma-Aldrich (Gillingham, UK) unless otherwise stated. All reagents were commercially available and used without further purification. Concentrated hydrochloric acid (37%, BP) and absolute ethanol were obtained from Merck Millipore (Nottingham, UK). Sodium chloride 0.9% for injection was obtained from Braun (Hessen, Germany). C18 light sample preparation cartridges (Waters, Elstree, UK) were conditioned with ethanol (10 ml), followed by water (10 ml) and air (10 ml). Accell Plus CM Plus cartridges (Waters, Elstree, UK) were conditioned with water (10 ml) and air (10 ml). Chromafix PS-OH^−^ cartridges (800 mg, Macherey-Nagel, Germany) were conditioned with 2 M HCl (20 ml) and air (20 ml). Vygon lines and connectors were obtained from Lectro-cath Vygon (Swindon, UK). Unless otherwise stated, TraceSELECT water for trace analysis (Fluka) was used throughout. Data were exported and statistical analyses were performed using GraphPad Prism (v.9.0). All parameters were analysed by unpaired t-test, *, *P* < 0.05; **, *P* < 0.01; ***, *P* < 0.001; ****, *P* < 0.0001.

### Production of ^62^Zn

A copper foil target (thickness 0.5 mm, diameter 1 cm, purity: 99.9%, purchased from Goodfellow Cambridge Ltd) was irradiated using an MC40 cyclotron (27 MeV protons, 35 µA for 9 h) at the School of Physics and Astronomy, University of Birmingham. Approximately 20 h after the end of bombardment, during which period radioimpurities such as ^61^Cu (t_1/2_ = 3.3 h) decayed substantially, the irradiated foil was transported to King's College London for extraction and purification of ^62^Zn as described below.

### Purification of ^62^Zn and preparation of ^62^Zn/^62^Cu generator

Purification and preparation of the ^62^Zn/^62^Cu generator was performed in a heavily shielded hot cell. The irradiated copper foil (∼0.7 g) was placed in a shielded 250 ml glass bottle containing hydrogen peroxide (25 ml, 30%, Fluka) and a magnetic stirrer bar on a magnetic stirrer under a fume extractor (see Fig. S1). Concentrated HCl (25 ml, 37%), followed by water (50 ml), was added to the bottle at a flow rate of 3 ml/min using a Watson Marlow peristaltic pump. The mixture was stirred using the magnetic stirrer throughout the procedure. Upon complete dissolution of the target, the blue-green mixture was passed through a shielded pre-conditioned strong anion exchange PS-OH^−^ cartridge driven by the negative pressure generated by a vacuum pump, and the eluate collected in a shielded waste bottle. The PS-OH^−^ cartridge was washed with 2 M HCl (80 ml, *via* tube 1, Fig. S1) to remove the copper, followed by absolute ethanol (50 ml, through tube 1, Fig. S1) to remove excess acid remaining on the cartridge. The ^62^Zn/^62^Cu generator was prepared by eluting ^62^Zn from the PS-OH^−^ cartridge with water (20 ml) and trapping it on a shielded, pre-conditioned hydrophilic cation exchange Sep-Pak Accell Plus CM Plus cartridge, while collecting the eluate in the shielded waste bottle. To remove all the radioactive and non-radioactive copper impurities from the cation exchange cartridge, glycine solution (105 ml, 200 mM in water) was passed through the cartridge into the waste bottle. At this stage, after a suitable induction period (∼20 min) to allow ^62^Zn to decay, ^62^Cu can be eluted repeatedly from the generator with further glycine washes and can be used to label a wide range of ^62^Cu radiopharmaceuticals. Elution details for ^62^Cu and subsequent radiolabelling of an exemplar radiotracer, [^62^Cu]Cu-ATSM, can be found in the supplementary information.

### Elution of ^62^Zn from the ^62^Zn/^62^Cu generator

Once immediate needs for ^62^Cu elution had been met, ^62^Zn was eluted from the generator with 4% trisodium citrate (1 ml, 136 mM in water, pH 6). Initially, this was used without further modification for *in vivo* studies similar to those reported for ^63^Zn by DeGrado *et al*.^25^^,^^26^ However, the high concentration of citrate was not tolerated well by mice. Therefore, for *in vivo* studies [^62^Zn]Zn-citrate solution (100 MBq, 100 μl) was diluted 16-fold in saline to give a ^62^Zn solution with a final citrate concentration of 0.25% (8.5 mM), well below the tolerated limit in mice, and a volume of 1.6 ml.

### Quality control of ^62^Zn

Verification of the radionuclidic purity was determined by gamma spectrometry using a ORTEC GEM Series High-Purity Germanium (HPGe) Coaxial Detector System coupled to a DSPEC jr 2.0 Digital Gamma-Ray Spectrometer. This system was calibrated in-house for energy and efficiency. Spectra were displayed and analysed with ORTEC GammaVision software (Version 6.01).

### Production of ^64^Cu

^64^Cu was produced as previously reported^35^ in the form of copper-64 chloride ([^64^Cu]CuCl_2_) in 0.1 M HCl solution. [^64^Cu]CuCl_2_ (∼100 MBq, 200 μl) was concentrated to dryness under N_2_ at 100°C for 10 min and the residue dissolved in 0.25% trisodium citrate (1 ml) to give [^64^Cu]Cu-citrate at a final pH of 6.

### PET/CT imaging

PET imaging was performed using a nanoScan-PET/CT (Mediso Medical Imaging Systems, Budapest, Hungary) system operating in list mode using 400–600 keV energy window and coincidence window of 1:3. CT scans were acquired for anatomical reference (55 keV X-ray, exposure time 1000 ms and 360 projections and pitch 1). PET projection data were reconstructed using the Tera-tomo® software package—a Monte Carlo-based fully 3D iterative algorithm with four iterations, six subsets, and 0.4 mm isotropic voxel size; corrections for attenuation, scatter, and dead-time were enabled. The data were then visualised and quantified using VivoQuant© (InviCro, Boston, USA) software. For 60-min scans, data were re-binned and reconstructed into a series of 1-min time frames for the first 5 min, 5-min time frames for the next 25 min and then 10-min time frames for the remainder of the 60-min scan period. For the 4 and 24 h scans, static 1-h images were reconstructed. For *in vivo* PET quantification, regions of interest (ROIs) were manually drawn over the brain, heart, liver, kidney, bladder, and thigh muscle. The CT images were used to define the boundaries of the organs. Given the large spillover of signal associated with ^62^Zn-PET between ROIs, care was taken to prevent this confounding organ uptake by avoiding regions where organs came into close proximity such as the liver and the apex of the heart. Time-activity curves were generated and expressed as percentage injected dose per gram of tissue (%ID/g). Area under the curve (AUC) for the regional TACs from 0 to 30 min (AUC_0-30 min_) and 30 to 60 min (AUC_30-60 min_) were calculated and compared between the radiometals.

### PET imaging phantoms with ^62^Zn, ^62^Cu, ^64^Cu, and ^18^F

A NEMA-NU4 image quality (IQ) phantom (Fig. S5) was filled and imaged with ^62^Zn/^62^Cu (7.5 MBq), ^62^Cu (5.1 MBq), and ^18^F (3.7 MBq). This phantom has compartments for investigating the IQ metrics of noise, activity concentration recovery coefficient (RC, the ratio of activity concentration in small volumes to that of a uniform region), and count spillover into enclosed chambers filled with air or nonradioactive water. A PET scan was acquired for 20 min followed by a CT scan**.** Image uniformity was expressed as the percentage standard deviation (% STD) of radioactivity observed in an ROI accounting for 75% of the active diameter drawn in the centre of the fillable region of the phantom. To evaluate the RCs and their standard deviations (RCSTD), the concentrations of radioactivity at six transaxial slices were averaged for each of the five fillable rods and divided by the mean total phantom activity concentration. Spillover ratios (SOR) were determined by measuring the apparent radioactivity in the non-radioactive water (SOR_water_) and air-filled (SOR_air_) chambers, divided by the mean total phantom activity concentration. Full width at half maximum (FWHM) was estimated by drawing several lines through a single point source from a 10 μl capillary tube (Jaytec, CAP-TF-10) and measuring the average distribution function. A second phantom, the Derenzo phantom, was used to visualise and compare spatial resolution of several different radionuclides by acquiring a 20-min PET scan followed by a CT scan. A similar radioactivity concentration was maintained for all PET radionuclides (∼1.5 MBq/ml).

### *In vivo* PET imaging with [^62^Zn]Zn-citrate and [^64^Cu]Cu-citrate

All animal experiments were performed in accordance with the Animals (Scientific Procedures) Act, 1986 with protocols approved by the Animal Welfare and Ethical Review Body for King's College London (St Thomas’ Campus). Female BALB/c mice (9–11 weeks of age, *n* = 3–4 animals/group, Charles River Laboratories) were anaesthetised by isoflurane inhalation (3%, Animalcare, York, UK, in O_2_), cannulated via a tail vein and placed prone on the scan bed. A bolus of radiotracer (∼5 MBq, 150 μl) was then administered i.v. *via* a tail vein cannula. Animals were maintained under isoflurane anaesthesia (Isocare®, 1.5–2% in O_2_) at 37 °C, and vital signs monitored throughout the scan. A dynamic PET scan was continuously acquired from 0 min post-injection (p.i.) for 1 h followed by a 10-min CT scan. Animals were then allowed to recover from anaesthesia and were re-anaesthetised and re-scanned at 4 h and 24 h p.i. for 60 min. This scanning protocol was performed for [^62^Zn]Zn-citrate and [^64^Cu]Cu-citrate. An additional cohort of female BALB/c mice (9–11 weeks of age, *n* = 3) were fasted for 12–16 h, scanned dynamically for 60 min with PET after i.v. injection of [^62^Zn]Zn-citrate, followed by a 10-min CT scan.

### *Ex vivo* biodistribution

*Ex vivo* biodistribution studies were performed in female (*n* = 4) and male (*n* = 4) BALB/c mice (9–11 weeks old) following an i.v. injection of [^62^Zn]Zn-citrate (∼0.7 MBq, 150 μl) or [^64^Cu]Cu-citrate (∼0.7 MBq, 150 μl) at both 1 h and 24 h p.i. The 24 h cohorts of mice in the female group were culled following the imaging protocol described above for both radiometals. The fasted cohort of mice was also culled for *ex vivo* biodistribution following the imaging protocol described above. All mice were culled *via* cervical dislocation, and tissues of interest were collected. All tissues were washed with phosphate-buffered saline (Sigma, 806552), blotted dry, weighed, and then counted using a gamma counter (1282 Compugamma; LKB, window set to channels 175–220; for the energy profiles see Fig. S4). *Ex vivo* biodistribution data were presented as %ID/g, where ID represents the total activity of all body parts excluding the tail.

## Results

### Production and purification of a ^62^Zn/^62^Cu generator

^62^Zn was produced using a high purity natural copper foil target via the ^63^Cu(p,2n)^62 ^Zn nuclear reaction. A saturated yield of approximately 1.5 GBq/μA was achieved; 9 h bombardment at 35 μA produced approximately 28 GBq of ^62^Zn at the end of bombardment. Radioactive decay over 20 h left 6 GBq; the target was then shipped to King's College London for purification. The irradiated target material was dissolved in hydrogen peroxide and hydrochloric acid and purified by a conventional method described previously by Fukumura *et al*. to produce a hydrophilic cation exchange Sep-Pak Accell Plus CM Plus cartridge loaded with ^62^Zn.^33^ Glycine (200 mM) was used to elute the radioactive and non-radioactive copper from the ^62^Zn/^62^Cu generator column. After 60 min, the generator had regenerated and ^62^Cu was eluted with glycine. The eluted [^62^Cu]Cu-glycine solution was then used to produce [^62^Cu]Cu-ATSM to a GMP standard, with radionuclidic purity > 99% and radiochemical purity > 97% by radio--high performance liquid chromatography analysis (Fig. S3), described here as an exemplar as proof of principle for the use of a ^62^Zn/^62^Cu generator to synthesise ^62^Cu-labelled bis(thiosemicarbazone) radiopharmaceuticals for clinical use. The application of these ligands and others in PET has been summarised previously.^34^^,^^36–39^

After multiple elutions to produce ^62^Cu radiopharmaceuticals, or simply to remove the ^62^Cu if it was not required, the remaining parent ^62^Zn could then be eluted with trisodium citrate (1 ml, 136 mM in water, pH 6) providing [^62^Zn]Zn-citrate (∼300 MBq), with 99.6% radionuclidic purity by HPGe γ spectrometry (characteristic peaks of ^62^Zn at 549 keV and 596 keV; 0.4% ^60^Co, t_1/2_ = 5.3 years; Fig. S2) and overall purification yield of 73.0 ± 8.2% (*n* = 3), which could then be diluted in saline ready for i.v. administration to evaluate the potential use of ^62^Zn for PET imaging of zinc trafficking.

### PET imaging phantoms with ^62^Zn, ^62^Cu, ^64^Cu, and ^18^F

**Fig. 1:**
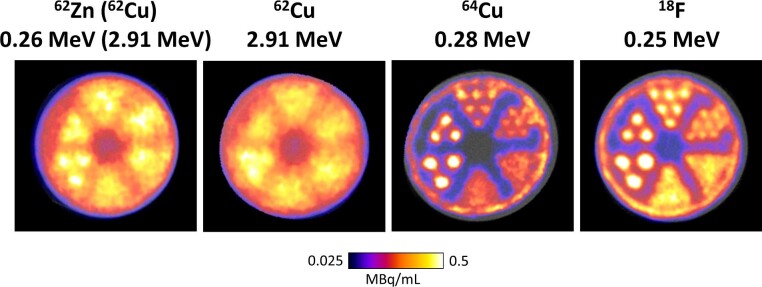
PET images of Derenzo imaging phantoms with various PET radionuclides using a preclinical nanoPET scanner. Mean positron energy in MeV is displayed under each radionuclide. Rod diameters descending clockwise from lower left are 4.8, 4, 3.2, 2.4, 1.6, and 1.2 mm.

PET images of the Derenzo phantom (Fig. 1) showed that capillary tubes filled with an equilibrated mixture of ^62^Zn/^62^Cu could not be resolved for the tube diameters tested. The largest rod diameter tested was 4.8 mm and thus spatial resolution was  ≥ 4.8 mm for ^62^Zn/^62^Cu. ^62^Cu alone displayed even poorer resolution, while the more popular copper radioisotope ^64^Cu and the ubiquitous clinical standard radionuclide ^18^F both displayed superior resolution, resolving diameters ≥ 2.4 mm. FWHM of 5.4 mm and 1.5 mm for ^62^Zn/^62^Cu and ^18^F respectively were estimated from a 10 μl capillary tube at centre of field of view. Industry standard NEMA-NU4 IQ phantoms highlighted that both ^62^Zn/^62^Cu and ^62^Cu had ∼3-fold greater spillover ratio (SOR) in air than ^18^F, but their SOR values were similar in water (Fig. S6). Additionally, %SD was 1.7-fold and 2-fold higher for ^62^Zn/^62^Cu and ^62^Cu respectively compared to ^18^F indicating poorer image uniformity for these radionuclides.

### ^62^Zn-PET in healthy mice compared with a ^64^Cu control

**Fig. 2:**
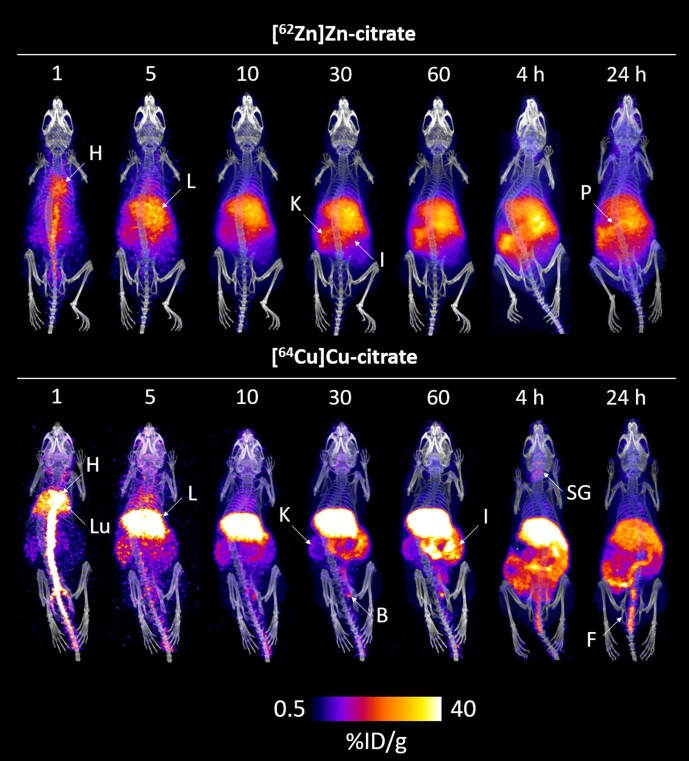
Dynamic PET/CT imaging of healthy mice after i.v. injection of [^62^Zn]Zn-citrate and [^64^Cu]Cu-citrate. Representative time course PET/CT maximum intensity projections of [^62^Zn]Zn-citrate (top) and [^64^Cu]Cu-citrate (bottom) post i.v. administration in female BALB/c mice (9–11 weeks old). (B = bladder, F = faeces, H = heart, I = intestines, K = kidney, L = liver, Lu = lungs, P = pancreas, and SG = salivary glands).

The biodistribution of [^62^Zn]Zn-citrate was investigated *in vivo* with PET imaging in female BALB/c mice at 0–1, 4, and 24 h p.i. (Fig. 2). The whole-body biodistribution generated from PET was then compared with [^64^Cu]Cu-citrate as a control to help distinguish the biodistribution of ^62^Zn from that of its daughter ^62^Cu present at the time of injection.

In mice given ^62^Zn, the majority of positron emissions at 1 h p.i. were observed from the heart, liver, kidneys, and intestines. Excretion was evident primarily *via* the hepatobiliary system. The biodistribution remained similar at 4 h, with decay-corrected activity gradually diminishing in organs over the next 20 h. By 24 h, as activity was excreted from the abdominal organs, the high radioactivity that had been present in pancreas but not readily discernible because of high abdominal background was more clearly evident. This biodistribution behaviour differed significantly at all imaging time points investigated from that of ^64^Cu injected in similar citrate buffer (as determined by PET) which at 4 and 24 h p.i. showed high activity in the liver, intestines, salivary glands, and kidneys, and elimination predominantly in the faeces and to a lesser extent in urine at early time points, but no obvious uptake in pancreas.

As in the phantom studies, the resolution of the mouse ^62^Zn/^62^Cu PET scans was inferior to that obtained with ^64^Cu. The 98% positron fraction of the daughter ^62^Cu (compared to just 8.2% with ^62^Zn) means ^62^Cu contributes most of the signal observed, and its high positron energy (2.91 MeV for ^62^Cu and 0.26 MeV for ^62^Zn, c.f. 0.27 MeV for ^64^Cu) causes relatively poor spatial resolution. These observations match those from ^62^Cu-PET investigations in the literature.^36^^,^^40^

**Fig. 3:**
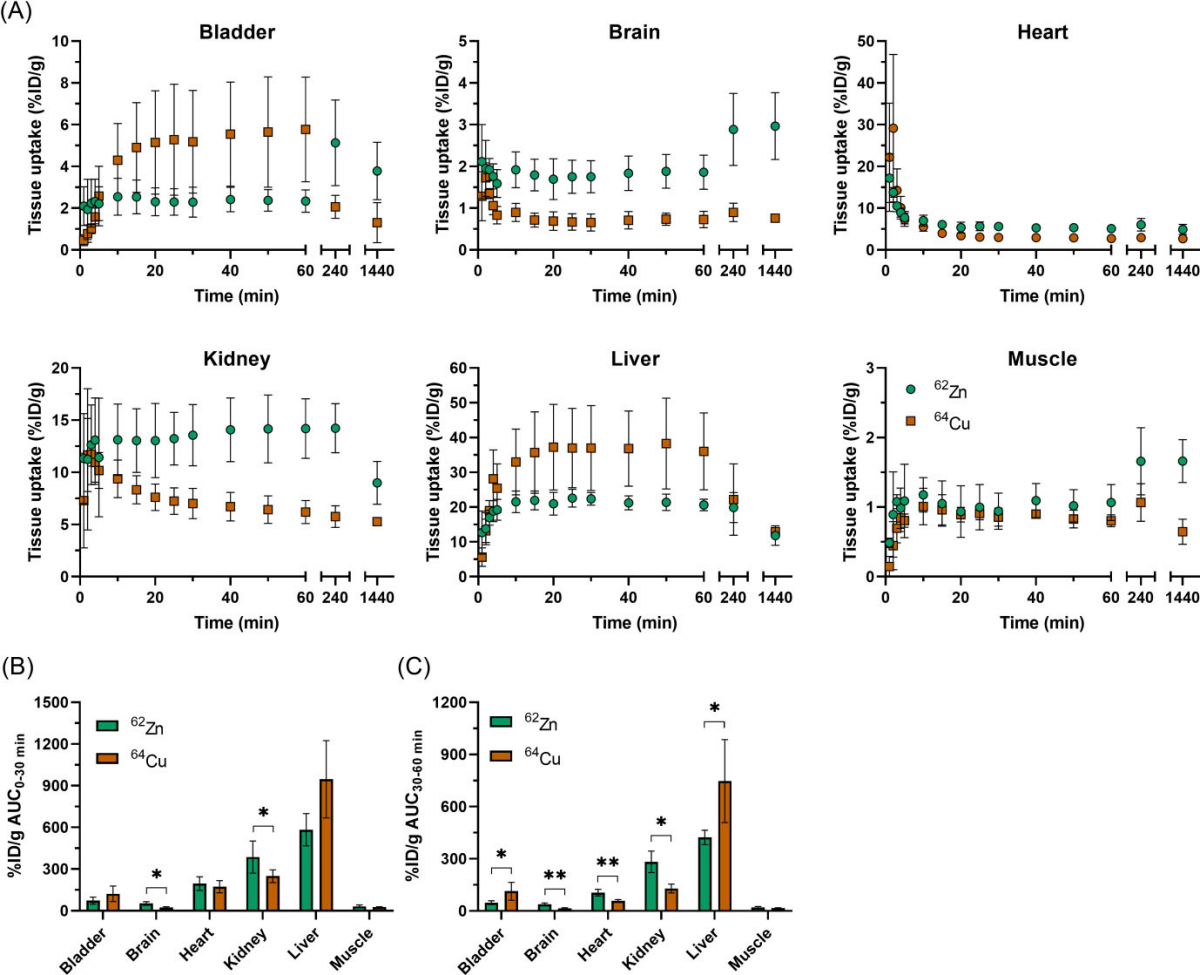
^62^Zn and ^64^Cu display different *in vivo* behaviours. (A) Time-activity concentration curves (TACs) for [^62^Zn]Zn-citrate (●) and [^64^Cu]Cu-citrate (■) in major organs of interest. (B and C) Area under the time activity curve (AUC) for tissues at 0–30 min (B) and 30–60 min post injection (C). **, *P* < 0.005. Error bars represent 1 s.d. from the mean value (*n* = 3–4 animals) and in some cases are smaller than the symbols.

To study the dynamic trafficking in more detail, PET data were segmented into bins to provide kinetic information on tissue uptake and clearance. Time-activity curves generated from the dynamic PET data are summarised in Fig. 3. Again, ^62^Zn and ^64^Cu differed substantially. ^64^Cu demonstrated an initial increase of activity in the kidneys in the first 2–3 min, followed by a decrease as radioactivity cleared to the urinary bladder. In contrast, after administration of ^62^Zn, while there was an initial accumulation of activity in the kidneys, this plateaued after 5 min and the majority did not clear to the bladder. Bladder, heart, liver, and muscle uptake over the first 5 min remained similar for both radiometals. However, a growing contribution from ^62^Zn with time, as well as distinct differences between copper and zinc handling, resulted in significant differences in uptake between radiometals. Uptake in the liver for both radionuclides plateaued after 10 min, and was significantly higher for ^64^Cu than for ^62^Zn (AUC_30-60 min_ = 747.1 c.f. 423.4 respectively; *P* = 0.0405). ^64^Cu however showed faster clearance from the liver with a 39% reduction in activity from 1 to 4 h p.i. (36.1 ± 11.1 vs 24.6 ± 8.4%ID/g, respectively), compared to just 4% for ^62^Zn (20.6 ± 1.7 vs 19.9 ± 4.3%ID/g, respectively). A gradual decline in liver uptake was then seen over 24 h for both radiometals. In the first few minutes following i.v. administration, there was an exponential clearance of both radiometals from blood with an initial half-life of < 2 min followed by a phase in which clearance was too slow for estimation of the half-life up to 60 min. ^62^Zn uptake in the heart was greater than that of ^64^Cu (AUC_30__–__60 min _= 105.1 for ^62^Zn c.f. 57.5 for ^64^Cu, respectively; *P* = 0.0098). Uptake in the brain was low but detectable for both radiometals; however, ^62^Zn was higher (AUC_30-60 min_ = 37.3 for ^62^Zn c.f. 14.5 for ^64^Cu, respectively, *P* = 0.0067). Brain ^62^Zn uptake increased 1.6-fold from 1 to 24 h p.i. (1.86 ± 0.41* *vs 3.00 ± 0.80%ID/g, *P* = 0.0606) but brain ^64^Cu at 24 h remained the same as at 1 h (0.81 ± 0.09 vs 0.73 ± 0.20%ID/g, *P* = 0.5422). Given the combination of high spillover from surrounding abdominal organs, as well as the ill-defined, diffuse anatomy of the mouse pancreas which cannot be visualised with CT without exogenous contrast agents, quantifying pancreas uptake from the PET images was not possible and pancreatic activity was determined by *ex vivo* gamma counting of dissected organs.

### *Ex vivo* biodistribution

**Fig. 4:**
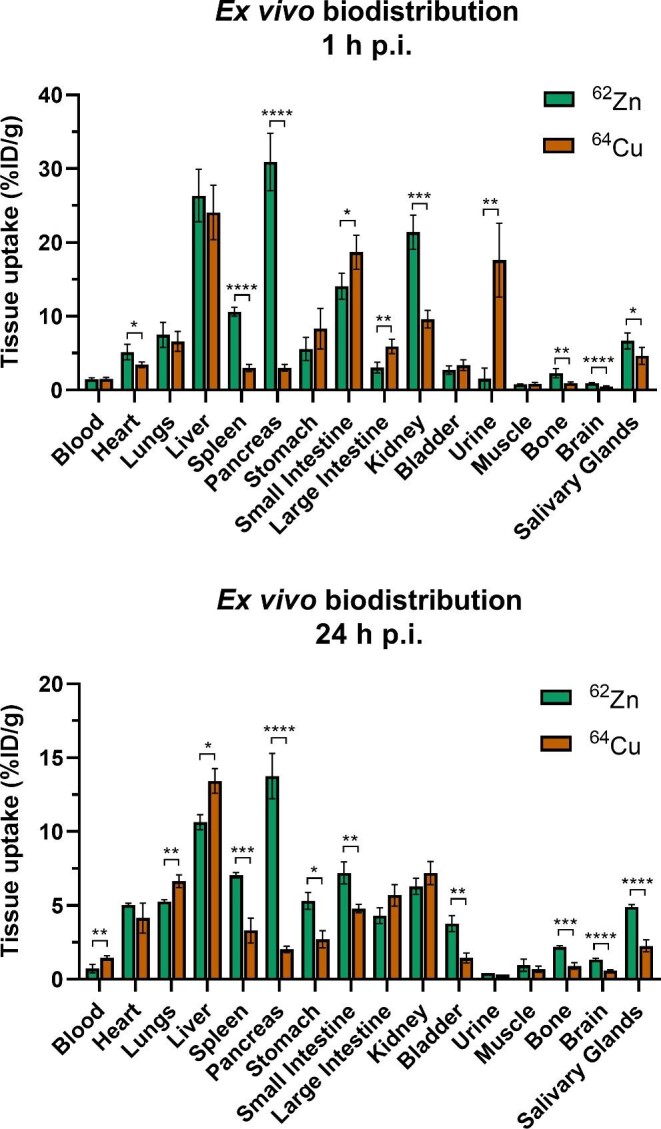
^62^Zn demonstrates significant differences in biodistribution compared to ^64^Cu. *Ex vivo* biodistribution of [^62^Zn]Zn-citrate and [^64^Cu]Cu-citrate in female BALB/c mice (*n* = 4) at 1 and 24 h after i.v. administration. Graphs represent mean ± SD. Comparisons were analysed for significance using an unpaired t-test, *, *P* < 0.05; **, *P* < 0.01; ***, *P* < 0.001; ****, *P* < 0.0001.

The *ex vivo* biodistribution of [^62^Zn]Zn-citrate and [^64^Cu]Cu-citrate at 1 and 24 h after i.v. injection was evaluated in female (Fig. 4) and male mice (Figs. S8 and S9) by weighing and gamma counting dissected organs. The results were consistent with the PET/CT images in all organs. [^62^Zn]Zn-citrate showed significantly (10-fold) higher pancreatic uptake compared to [^64^Cu]Cu-citrate at 1 h p.i. (30.90 ± 3.88 vs 3.03 ± 0.46%ID/g, *P* < 0.0001). Although there was some (∼60%) clearance of ^62^Zn from pancreas by 24 h, the high ^62^Zn:^64^Cu ratio in pancreas persisted. Uptake in the heart, spleen, kidney, bone and salivary glands was also significantly higher for ^62^Zn than ^64^Cu at 1 h p.i. Differences in bladder activity observed in the PET images are not evident in the *ex vivo* biodistribution at 1 h; however, significantly lower radioactivity was observed in the urine with [^62^Zn]Zn-citrate compared to [^64^Cu]Cu-citrate at 1 h p.i. (1.54 ± 1.46 vs 17.61 ± 5.00%ID/g, respectively, *P* = 0.0032). Given the time delay between collecting the organs and measuring them on the gamma counter, any ^62^Cu present in the urine at the time of culling would have since decayed. As observed during PET quantification, brain accumulation of ^62^Zn increased at 24 h compared to 1 h p.i. (1.39 ± 0.13 vs 0.95 ± 0.07%ID/g respectively, *P* = 0.0009). Additionally, ^62^Zn uptake in the brain was significantly higher than that of ^64^Cu at 1 h (0.95 ± 0.07 vs 0.57 ± 0.07%ID/g, respectively, *P* < 0.0001) and 24 h p.i. (1.39 ± 0.13 vs 0.53 ± 0.06%ID/g respectively, *P* < 0.0001). In male mice, ^62^Zn uptake increased 1.7-fold in the prostate and seminal vesicles from 1 to 24 h p.i. (1.19 ± 0.18 vs 1.98 ± 0.29%ID/g, respectively, *P* = 0.0035, Fig. S8) and was higher at both time points than ^64^Cu which decreased over this interval. Differences in biodistribution between genders were also observed (Fig. S10 for ^62^Zn, Fig. S11 for ^64^Cu). Higher brain, spleen, liver, and kidney uptake of ^62^Zn in female than in male mice was observed. Compared to males, greater accumulation of ^64^Cu was seen at 1 h p.i. in the stomach, intestines, kidneys, and brain of female mice. These gender-specific differences remained at 24 h. Reduced hepatic ^64^Cu uptake in female mice was observed at 1 and 24 h p.i., but was only statistically significant at 24 h p.i.

The effect of fasting on ^62^Zn biodistribution in mice over 1 h was investigated to see if biliary clearance into intestines is reduced. Reduced activity in the intestines would be beneficial for clinical PET investigations in the abdominal region. However, fasting animals for 14–18 h prior to ^62^Zn injection had no significant impact on the delivery of ^62^Zn from the blood to organs, or its retention in organs, including the liver, over a period of 1 h (see Fig. S12 for *ex vivo* biodistribution).

## Discussion

A ^62^Zn/^62^Cu generator provides practical and reproducible access to short-lived ^62^Cu radiopharmaceuticals such as bis(thiosemicarbazone) complexes. Over the years, the properties of bis(thiosemicarbazone) complexes have been fine-tuned to provide a family of radiotracers, some designed as perfusion agents and others that provide a hypoxia-dependent PET signal. With the advent of total-body PET,^41^ the production of short-lived radiotracers, such as ^62^Cu, will bring new possibilities as they can be ‘multiplexed’ with other PET tracers to provide a more comprehensive assessment of diseases such as cancer. Similar generator-based systems to the one described here (Supplementary methods) have been used for many years to produce ^62^Cu,^31–34^ but little biological research has been performed with the parent radionuclide ^62^Zn. We have shown here that once the generator has met the demand for ^62^Cu production, elution of ^62^Zn can be reliably performed using trisodium citrate, producing a solution that can be diluted with saline and used directly for *in vivo* studies, without additional purification steps. Modifications to elute the ^62^Zn with different buffers is in principle possible to yield ^62^Zn in chemical forms other than citrate, but we have not yet evaluated this potential. Here, we aimed to investigate the whole-body biodistribution of [^62^Zn]Zn-citrate as a preliminary evaluation of its potential for imaging zinc trafficking *in vivo*, particularly over longer time periods than are possible with the previously reported studies with ^63^Zn PET, and to assess the extent to which interpretation of images would be confounded by the *in vivo* conversion to ^62^Cu, from which the vast majority (>90%) of positrons would originate. Our hypothesis was that, to a reasonable approximation, the PET images would reflect the biodistribution of ^62^Zn despite the PET signal originating largely from the daughter ^62^Cu. Two extreme scenarios can be envisaged. In one extreme, pure ^62^Zn is injected immediately after elution from the generator and before significant decay to ^62^Cu. This, of course, is likely to be unattainable in practice because radioactive equilibrium will be approached within a few minutes of the last elution of ^62^Cu. In this first scenario, the rapid initial deposition of radioactivity from blood into tissues means that the PET image reflects largely zinc. At the other extreme (more realistic in practice), an equilibrium mixture of ^62^Zn and ^62^Cu is injected. The low positron branching ratio (8.2%) and longer half-life (9.3 h) of ^62^Zn compared to ^62^Cu (98%, 9.7 min) means that >90% of the positrons from this mixture originate from ^62^Cu. Immediately after injection, the observed biodistribution therefore initially reflects the injected ^62^Cu, with a growing contribution from ^62^Zn over the next few minutes. Once the injected ^62^Cu has decayed (a process that is essentially complete by 1 h p.i., corresponding to six half-lives), the signal thereafter comes either directly from ^62^Zn decay (8.2%) or from ^62^Cu that has been formed *in vivo*, and the latter radioactivity has on average spent >98% of its existence *in vivo* as ^62^Zn (a consequence of the ratio of the half-lives of 9.3 h and 9.7 min). The rapid clearance of zinc from the blood,^25^^,^^26^ as seen with other essential metals such as copper^22^^,^^23^^,^^42^ and manganese,^43^^,^^44^ means that the majority of ^62^Zn decay would take place once it reached the tissue of interest. Only if the decay of ^62^Zn that has been deposited in its target tissue causes rapid redistribution of the daughter ^62^Cu (within a few minutes) and release from that tissue, will the image fail to reflect zinc trafficking to a reasonable approximation. We therefore proposed that over a period of up to 2 days, and with the exception of the first hour p.i., ^62^Zn is a useful option for studying zinc trafficking *in vivo*.

The biodistribution and imaging data described in this manuscript are consistent with this rationale. The *ex vivo* biodistribution of ^62^Zn (and its *in vivo* generated daughter ^62^Cu) at 1 and 24 h p.i. was compared with that of ^64^Cu (which is free of radioactive parent or daughter complications) in healthy mice, showing a significant difference in uptake of ^62^Zn and ^64^Cu in the liver, kidney, pancreas, salivary glands, spleen, and prostate/seminal vesicles. This is the first time, to our knowledge, that the biodistribution of ^62^Zn and ^64^Cu has been directly compared *in vivo* with PET. The profound differences seen between the ^62^Zn and ^64^Cu images provide evidence that the PET images post 1 h (and even pre-1 h to an extent) and biodistribution data reflect trafficking mainly of zinc and not copper.

Having drawn this conclusion, we are in a position to use our data to describe the trafficking of i.v.-injected zinc over a longer time period than was previously possible. The biodistribution of ^62^Zn is consistent with the previously observed biodistribution of ^63^Zn reported by DeGrado *et al*.^25^ with predominant uptake being observed in the pancreas, liver, kidney, intestines, and spleen. The results are in agreement with the essential role of zinc in the pancreas, where it mediates processing, storage, secretion, and action of insulin in pancreatic β cells,^45^^,^^46^ and in the prostate, where zinc inhibits aconitase resulting in the accumulation of citrate which may serve as a zinc ligand in prostatic secretion and in seminal fluid.^4^^,^^47^ What remains unknown is whether or not the acute delivery and distribution of radiometals matches bulk metal levels in the body accumulated over the subject's lifetime; this depends upon how rapidly the long- and short-term zinc pools (which will have both labile and inert components) equilibrate. Additional comparisons to ICP-MS measurements of total tissue zinc and copper content will be required in the future.

The major benefit of ^62^Zn compared to ^63^Zn is the ability to study zinc trafficking over several hours or days instead of 1–2 h. Imaging at 4 and 24 h p.i. showed excretion and redistribution that has not been observed previously with ^63^Zn, due to its short half-life. Notably, uptake in brain and prostate/seminal vesicles was significantly higher (∼2-fold) at 24 h compared to 1 h p.i. This indicates redistribution of zinc into these tissues from other organs over time. The activity in the majority of organs, particularly the liver and pancreas, decreases at 24 h but still remains prominent, suggesting some redistribution. Furthermore, significant radioactivity in the intestines suggests that the majority of Zn excreted from tissues is eliminated *via* faeces.

The dynamics of zinc trafficking has been modelled previously in rodents and humans^28^^,^^48^^,^^49^; however, the models were derived from studies where ^65^Zn was administered orally or subcutaneously, or used radioactive zinc in a form other than citrate. Intravenous administration as a delivery method bypasses some of the physiological metal trafficking routes, such as intestinal absorption and the first-pass liver uptake of radioactivity delivered by the hepatic portal vein. The long half-life of ^65^Zn allowed investigation of zinc kinetics for weeks.^50^ Oral administration resulted in lower %ID compared to our findings; this is expected because not all of the radioactivity is likely to be absorbed from the gut. Rapid kinetics of zinc uptake in tissues from the blood matched compartmental models derived from ^65^Zn biodistribution data. The large decrease of ^62^Zn observed in the liver, kidney, and pancreas at 24 h p.i. compared to 1 h p.i. agrees with turnover times previously reported. However, accurate turnover times for organs over a day, although feasible in principle with ^62^Zn, could not be determined from our preliminary data given the limited time points studied. Nevertheless, because of the availability and evident utility of ^62^Zn, PET now offers the opportunity to provide a comprehensive understanding of metal dynamics after various administration routes without the need to cull large numbers of groups of animals at predetermined time points.

A disadvantage of using ^62^Zn instead of ^63^Zn is that kinetics at early time points cannot be investigated quantitatively without correction by modelling the contribution of the daughter ^62^Cu. ^62^Cu present at the time of injection confounds early measurements (< 1 h). ^64^Cu data provides the information required to make this correction. Nevertheless, if blood dynamics or tumour kinetics over the first few minutes are needed, ^63^Zn is a much more suitable option. Also, the resolution of ^62^Zn/^62^Cu is poor in comparison to ^63^Zn, because of the high positron energy of ^62^Cu (2.91 MeV for ^62^Cu c.f. 0.99 MeV for ^63^Zn). This is a significant issue when imaging mice, as seen in Fig. 2, but is less problematic in humans. Post-processing methodologies such as image resolution recovery could be implemented here to improve the spatial resolution of ^62^Zn images, and are currently being validated. On the other hand, *ex vivo* biodistribution data are unaffected by the problem of spatial resolution, allowing us to quantitatively show similarity of ^62^Zn to ^63^Zn and their marked contrast to ^64^Cu—particularly in pancreas uptake and urinary excretion.

Interestingly, early urinary excretion was observed consistently with ^64^Cu but was much less with ^62^Zn. Kidney uptake was higher with ^62^Zn suggesting that the zinc is being reabsorbed and retained within the kidney rather than excreted. [^64^Cu]Cu-citrate displayed similar biodistribution to other ionic forms of copper (e.g. liver and intestinal uptake); however, renal excretion of ^64^Cu has not been reported as a major excretion pathway with other ionic forms of copper (dichloride and acetate).^22^^,^^24^^,^^51^ Citrate enhances early renal excretion, probably by forming a stronger complex with a more prolonged stability *in vivo* than the chloride or acetate forms*,* perhaps delaying transchelation to albumin sufficiently to allow glomerular filtration of the low-molecular weight copper complexes, whereas Cu administered as acetate or chloride very rapidly binds to albumin, which is not subject to glomerular filtration.^24^ This important observation demonstrates that radiometals administered in different weakly chelated forms can display different behaviour *in vivo*. Therefore, the choice of administration buffer should be carefully considered for the radiometal application. For example, ^64^CuCl_2_ has been used to image prostate cancer in clinical subjects^51^^,^^52^; in this context, [^64^Cu]Cu-citrate may not be an appropriate radiotracer for imaging prostate cancer as it could obscure the signal in the pelvic area due to urinary excretion. Establishing the effect different ionic speciation of radiometals has on their biodistribution is an important area for PET metallomics, as optimisation of the delivery of radiometals to tissues (tumours for example) could improve their diagnostic power.

Performing studies in both male and female mice was important to provide a base for future investigations in gender specific diseases, for example exploring the role of zinc as a biomarker in prostate cancer in men, or in breast cancer in women. The large body of evidence that supports the decline in endogenous zinc in prostate cancer compared to healthy prostate means that PET with radiozinc, as a largely diagnostic-focused tool in the clinic, has little benefit as radiozinc delivery is also likely to be reduced rather than increased in malignancy. This would lead to a hard-to-detect cold spot on a PET scan representing cancerous tissue, against a high background in surrounding healthy prostate tissue showing accumulation of radiozinc. The role of Zn in breast cancer is less clear than in prostate cancer but potentially as important. There is growing evidence that zinc is elevated in breast cancer (∼1.2- to 6.5-fold increase compared to stromal breast tissue).^53–57^ Moreover, zinc might be a useful biomarker for determining breast cancer subtype.^57^^,^^58^ Farquharson *et al*. demonstrated that oestrogen receptor positive (ER+) tumour samples had approximately 80% higher zinc concentrations than in oestrogen receptor negative (ER−) ones.^58^ This relationship could be studied further *in vitro* with radiozinc cell uptake studies and then in animals using PET imaging. Another exciting alternative is investigating the role of zinc in tamoxifen resistance. Taylor and co-workers have shown increased endogenous zinc in tamoxifen-resistant cells (using a Tam-R cell line established from MCF7 by chronic exposure to tamoxifen). The 2-fold increase in zinc was accompanied by a significant increase in expression of ZIP7—a zinc influx transporter thought to mediate uptake of zinc into the endoplasmic reticulum.^59^ Future experiments studying zinc in breast cancer with PET imaging might therefore be of significant value. Apart from breast cancer, imaging zinc could be useful for other disease states. Possible applications include imaging delivery and retention of zinc in diabetic pancreata, arthritic joints, Wilson's disease livers, and Alzheimer's disease brains.

Radionuclides, especially those with a long half-life (e.g., ^65^Zn), have long been important tools for metallomics research. Even without imaging, they have provided vital insight into metal dynamics from the cellular to the whole body level. However, without the advantages afforded by imaging, their use is limited; for example, large groups of animals must be culled at specific time points, each of which provides only a snapshot in time of metal distribution. Such studies are unfeasible in humans. PET metallomics as a non-invasive imaging paradigm aims to go beyond this, utilising positron-emitting radiometals to visualise metal kinetics and dynamics not just in selected organs but in all organs. The work described in this manuscript establishes ^62^Zn as a tool to study zinc trafficking dynamically on a whole-body level, and provides new data that goes beyond the early time points reported by DeGrado *et al*. for ^63^Zn.^25^^,^^26^ For example, the increased brain uptake at 24 h p.i. compared to 1 h p.i. may afford new insights into Alzheimer's disease that could not be studied previously with ^63^Zn.^26^ With expanding access to PET technology, ^62 ^Zn and ^63^Zn can be harnessed to study zinc *in vivo* with PET to answer questions such as: What is the role different transporters play in Zn uptake and efflux? What effect does the local expression of these transporters have on zinc handling *in vivo*? How effective are novel chelator therapies at removing zinc from tissues, such as brain in Alzheimer's disease? Is there undesirable redistribution of the zinc after chelation therapy for copper overload in Wilson's disease? With the wealth of literature available on intracellular zinc handling, and the diverse range of preclinical models of systemic and localised transporter deficiencies recently reported, these tools are poised to make significant impact. For their full value to be realised, a collaborative approach is needed, bringing together imaging scientists, metal biologists, and inorganic chemists to exploit radiometal PET for the study of metal trafficking and clinical applications in trace metal-related disease.

## Conclusions

The ability of ^62^Zn to image zinc trafficking on a whole-body scale *in vivo* has been demonstrated in this work. We have provided evidence that the PET images obtained after injection of ^62^Zn as its citrate complex reflect the trafficking *in vivo* primarily of zinc and not copper, despite potential complexity due to its *in vivo* decay to the positron emitter ^62^Cu. Similarly to ^63^Zn, which is free from such complications, ^62^Zn acutely localises to known zinc-rich organs such as the pancreas, kidney, prostate, and seminal vesicles. For example, [^62^Zn]Zn-citrate showed significantly higher (10-fold) pancreatic uptake compared to [^64^Cu]Cu-citrate at 1 h p.i. Although ^62^Cu present at the time of injection confounds early kinetic measurements in the first hour p.i., PET imaging with ^62^Zn provides an important quantitative tool that can be used to study zinc trafficking at later time points (up to 2 days), which was not possible with ^63^Zn (showing that liver, pancreas, and kidney uptake diminished, but brain uptake increased, at 24 h p.i.). Importantly, we have demonstrated new insights into how the body handles zinc differently compared to copper. Harnessing these PET tools will help in determining the role that metal trafficking plays in human health and disease and will be useful in preclinical models and human studies where metal homeostasis is dysregulated.

## Supplementary Material

mfac076_Supplemental_File

## Data Availability

The data underlying this article will be shared on reasonable request to the corresponding author.
