# Supplementary material for: Imaging zinc trafficking in vivo by positron emission tomography with zinc-62
Source: Metallomics. 2022 Oct 6;14(10):mfac076. doi: 10.1093/mtomcs/mfac076 (PMC9578021; doi:10.1093/mtomcs/mfac076)
Supplement: mfac076_Supplemental_File [file mfac076_supplemental_file.docx]

**Electronic supplementary information**

**Imaging zinc trafficking in vivo by positron emission tomography with zinc-62**

George Firth^a*^, Zilin Yu^a*^, Joanna J. Bartnicka^a^, David Parker^b^, Jana Kim^a^, Kavitha Sunassee^a^, Hannah E. Greenwood^a^, Fahad Al-Salamee^a^, Maite Jauregui-Osoro^a^, Alberto Di Pietro^a^, Joanna Guzman^a^, Philip J. Blower^a†^.

**Supplementary methods**

Elution of ^62^Cu and preparation of [^62^Cu]Cu-ATSM

Prior to eluting the generator, aqueous sodium acetate (100 µL, 0.25 M, pH 5) was dispensed into a shielded 10 mL glass vial, which was connected to the generator by piercing the rubber septum of the vial with a needle connected to the outlet of the generator (lead pot 3, **Fig. S1**). After 60 min of regeneration, the generator was eluted with aqueous glycine (3 mL, 200 mM, *via* tube 2). A solution of H_2_ATSM (ABX, Germany, 1 mg/mL) in DMSO was freshly prepared and 10 µL of this solution was immediately added to the vial. The reaction vial was agitated and kept at room temperature for 3 min, while the activity was measured in a dose calibrator (CRC-712M, Capintec), using the manufacturer’s 448 calibration factor for Cu-62. The solution was manually loaded onto a shielded, pre-conditioned C18 light cartridge (Waters, Elstree, UK) using a shielded syringe. The C18 cartridge was washed with saline (10 mL), before eluting the [^62^Cu]Cu-ATSM product with absolute ethanol (0.4 mL, Martindale Pharma, Romford), followed by saline (4.5 mL), into a nitrogen filled sterile vial (N46, GE Healthcare). The vial containing the product was then transferred to a remote sterile isolator, for filtering through a 0.22 µm filter (13 mm Millex GV, Millipore) and QC sampling. During the dispensing process, the sterilisation filter was contained within the isolator and was not exposed within the environment of the hot cell, ensuring final product sterility.

Quality control of [^62^Cu]Cu-ATSM

Radiochemical purity and identity of [^62^Cu]Cu-ATSM were checked by a HPLC system (1260, Agilent) with in-line UV-Vis and radioactivity detector, using a Zorbax SB-C18 column (4.6 x 150 mm, 5 µm) at flow rate of 1.0 mL/min with a water (solvent A)/methanol (solvent B) gradient as the mobile phase. The gradient started with 50% B, increasing to 90% B at 7.5 min before decreasing it to 50% B at 8 min and maintaining 50% B until the end of the run (13 min). The UV-Vis wavelength used during the analysis was 462 nm. The concentration of H_2_ATSM present in each batch of [^62^Cu]Cu-ATSM was reported as < 1.0 µg/mL or > 1.0 µg/mL depending on the HPLC UV peak area of the H_2_ATSM. The concentration of Cu-ATSM present in each batch of [^62^Cu]CuATSM was reported as < 0.2 µg/mL or > 0.2 µg/mL depending on the HPLC UV peak area of the Cu-ATSM peak. The pH of the [^62^Cu]CuATSM product was checked using pH paper (range 4.5 – 8.5). Quality control results are summarised in **Table S1** and **Fig. S3.**

Radioactivity

The radioactivity of [^62^Cu]Cu-glycine complex was measured after its elution from the generator. The radioactivity of the ^62^Cu-ATSM complex (activity for release) was measured after the QC sample had been taken (**Table S1**). Radioactivity was measured using a dose calibrator (CRC-712M dose calibrator, Capintec).

Visual inspection

The [^62^Cu]ATSM product was checked by visual inspection to ensure that it was clear and free from particles (**Table S1**).

Radionuclide purity

Verification of the radionuclidic purity was carried out via gamma spectrometry using an ORTEC GEM Series High-Purity Germanium (HPGe) Coaxial Detector System coupled to a DSPEC jr 2.0 Digital Gamma-Ray Spectrometer which had been calibrated in-house for energy and efficiency. The presence of the ^62^Zn and ^64^Cu contaminants in the [^62^Cu]Cu-ATSM final product was determined by measuring their 596 and 1345 keV γ lines, respectively. All spectra were displayed and analysed with ORTEC GammaVision software (Gamma Vision for Windows Model A66-B32, Version 6.01) (**Fig. S2** and **Table S1**).

Half-life measurements

Determination of the radioactive half-life of the [^62^Cu]Cu-ATSM was carried out by placing the product vial into the dose calibrator (CRC-712M dose calibrator, Capintec) and recording the activity measured by the calibrator over a period of 10 min as the product decayed (**Table S1**). The natural logarithm of each measured activity was plotted against the measurement time and a linear model fitted to the data using linear least squares regression. The negative gradient of the best fit line is the decay constant (*λ*) of the data, from which the half-life can be calculated according to

$$t_{\frac{1}{2}}= \frac{\ln(2)}{\lambda}$$

Endotoxin testing

[^62^Cu]Cu-ATSM batches were tested for endotoxin following the protocol for Limulus Amoebocyte Lysate (LAL) testing using the Endosafe-PTS portable system (Charles River, France) (**Table S1**). Maximum valid dilution (MVD) or minimum valid concentration (MVC) was calculated in order to know how much room for dilution there was for the drug product to be tested for endotoxin. To do this, Inhibition/Enhancement (I/E) was performed by running samples between undiluted and the MVD/MVC.

Filter integrity testing

The integrity of the filters used for the sterilisation of the [^62^Cu]Cu-ATSM batches was established by performing the bubble point test (**Table S1**).

Residual solvent analysis

[^62^Cu]Cu-ATSM batches were analysed for residual solvents (i.e. ethanol) and are summarised in **Table S1**. The analysis was performed by Butterworth Laboratories (Teddington, UK).

Sterility Bioburden

Sterility and bioburden testing was carried out by Wickham Laboratories (Hampshire, UK) for all [^62^Cu]Cu-ATSM batches manufactured (**Table S1**).

**Supplementary figures**


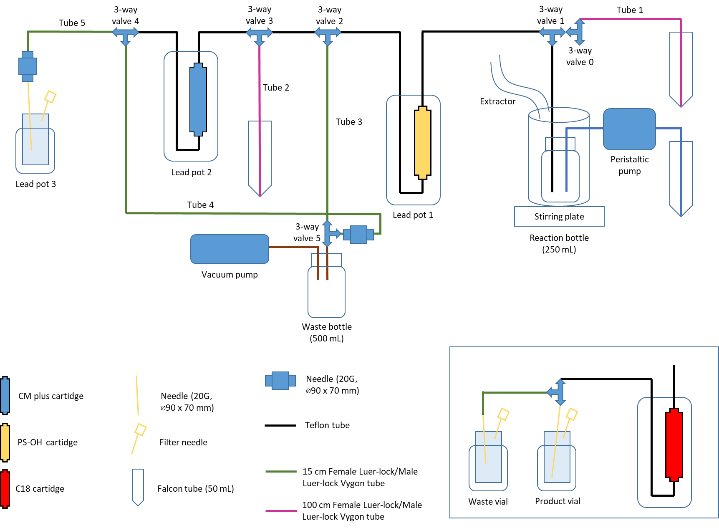


**Fig. S1 Schematic diagram of the system for target dissolution, Zn-62 separation and generator preparation.** The reaction bottle for dissolution of irradiated copper foil, PS-OH- cartridge, Accell Plus CM Plus cartridge and waste bottle were connected with disposable tubing and three-way-manual valves. Negative pressure generated by vacuum pump and waste bottle drove the target dissolution mixture, 2M HCl solution, low metal content water and glycine solution through cartridges at different stages and ended in a waste bottle. The system was situated in a ventilated hot cell, minimising the manual operation and radiation dose for the operator and increasing the repeatability of the generator preparation process.


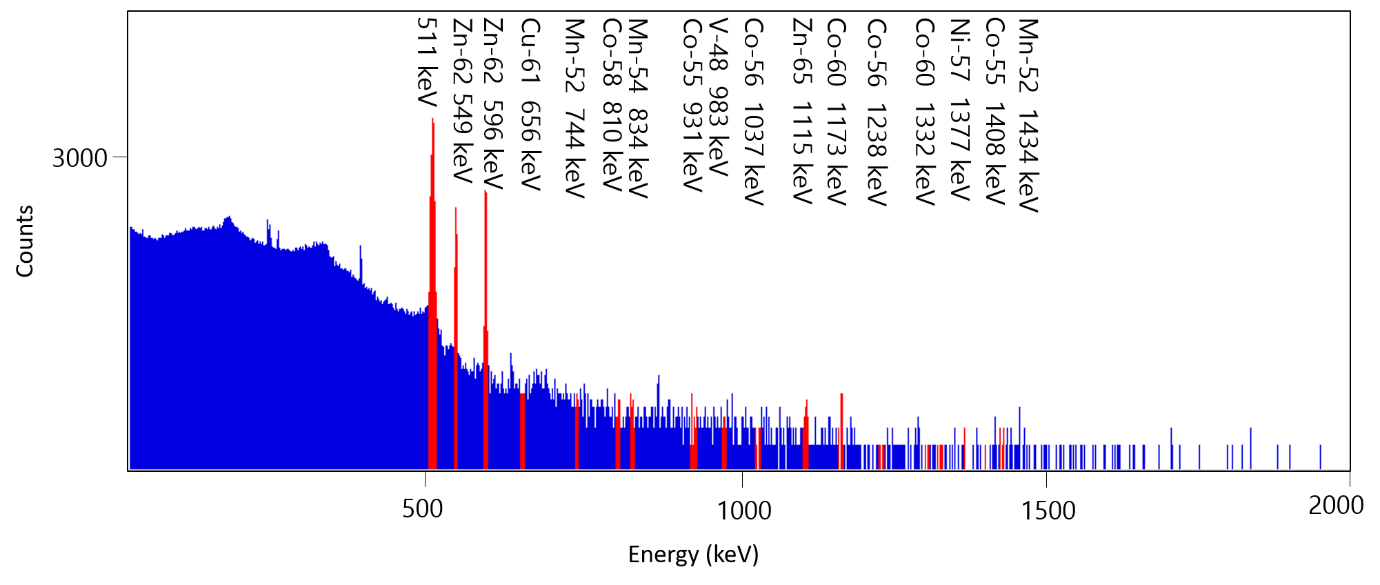


**Fig. S2 Representative gamma spectrum for ^62^Zn after purification (acquisition time 300 s).** The 511, 549 and 596 keV emissions from ^62^Zn are dominant among the background peaks.

**Table S1: [^62^Cu]Cu-ATSM production. 12 radiosyntheses were performed from 3 different ^62^Zn/^62^Cu generators.**

| **Test** | **Acceptance Criterion** | **Average Result** ± SD **(*n* = 12)** |
| --- | --- | --- |
| **Radiochemical Purity (%)** | > 95 | 97.8 ± 0.5 |
| **Radioactive Impurities (%)** | < 5 | Pass |
| **pH** | 4.5 - 8.0 | 6.8 ± 0.4 |
| **Volume (mL)** | 3 – 8 | 4.5 |
| **Appearance and Colour** | Clear and Particle free | Pass |
| **Stability (checked by HPLC)** | Stable | Pass |
| **Activity eluted from the generator (MBq)** | ≥ 1100 | 1887 ± 522 |
| **Activity of product for release (MBq)** | > 300 | 460 ± 124 |
| **Endotoxin Limit (Endosafe-PTS) (EU/mL)** | < 5 | < 1.00 |
| **H_2_ATSM Concentration (μg/mL)** | < 1 | < 1 |
| **CuATSM concentration (μg/mL)** | Report value | < 0.2 |
| **Filter integrity test (psi)** | Bubble point >50 | 56.0 ± 2.6 |
| **Sterility** | Sterile | Sterile |
| **Solvent analysis (% EtOH)** | < 10% Ethanol | 8.2 ± 0.5 |
| **Radionuclidic identity** | Half-life: 9.22-10.1 min | 9.65 ± 0.10 |
|  | Gamma: 0.511 MeV | 0.511 MeV |


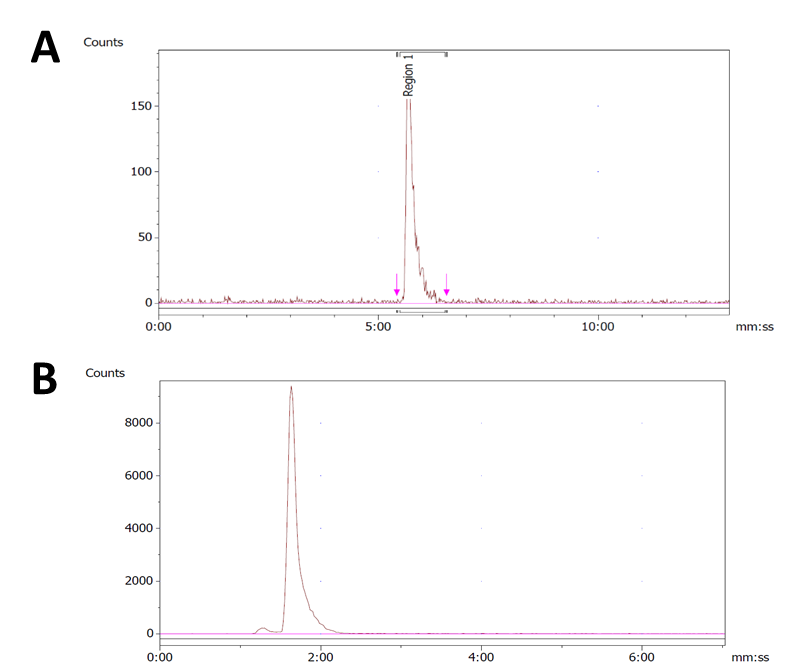


**Fig. S3 Representative HPLC chromatograms for [^62^Cu]Cu-ATSM radiolabelling.** A: Following C18 cartridge purification, a single peak was observed in the radiochromatogram consistent with the retention time of [^62^Cu]Cu-ATSM (retention time 5:45 minutes:seconds). B: The generator eluent [^62^Cu]Cu-glycine elutes earlier than [^62^Cu]Cu-ATSM with a retention time of 1:40 minutes:seconds. Column: Zorbax SB-C18 column (4.6 x 150 mm, 5 µm); solvent A: H2O (0.1% TFA), solvent B: MeCN (0.1% TFA); flow rate: 1 mL/min; UV detector: 462 nm; gradient: 50-90% B, 0-7.5 min; 50% B, 8 min; 50% B, 13 min.

**Fig. S4 Gamma spectra for ^62^Zn/^62^Cu mixture, ^62^Cu and ^64^Cu acquired by gamma counter.** Emissions from ^62^Zn/^62^Cu (top left), ^62^Cu (top right) and ^64^Cu (bottom left) across all energy windows detectable by the gamma-counter. The range of windows used to measure the presence of ^62^Zn/^62^Cu (175-220, 511 keV peak), ^62^Cu (175-220, 511 keV peak) and ^64^Cu (175-220, 511 keV peak) in samples are shown in green for Zn and brown for Cu.


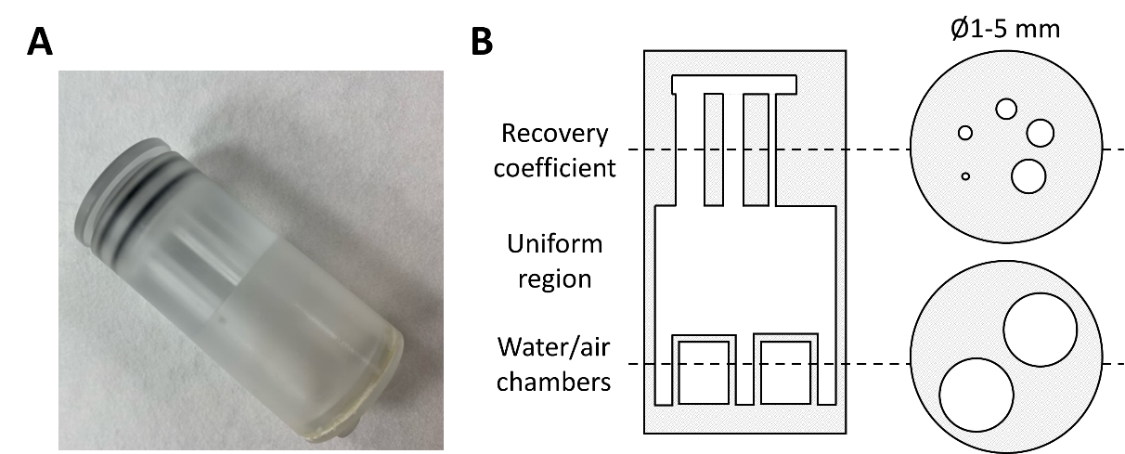


**Fig. S5 Industry standard NEMA NU-4 for assessing image quality.** NEMA NU-4 image quality (IQ) phantom (A) and a schematic of its different compartments (B). 5 fillable rods (diameter, 1, 2, 3, 4, and 5 mm) measure recovery coefficients, a homogeneous fillable region (uniform region) in the centre of the phantom measures uniformity and 2-chamber regions (air-and water-filled) measure spillover ratios.


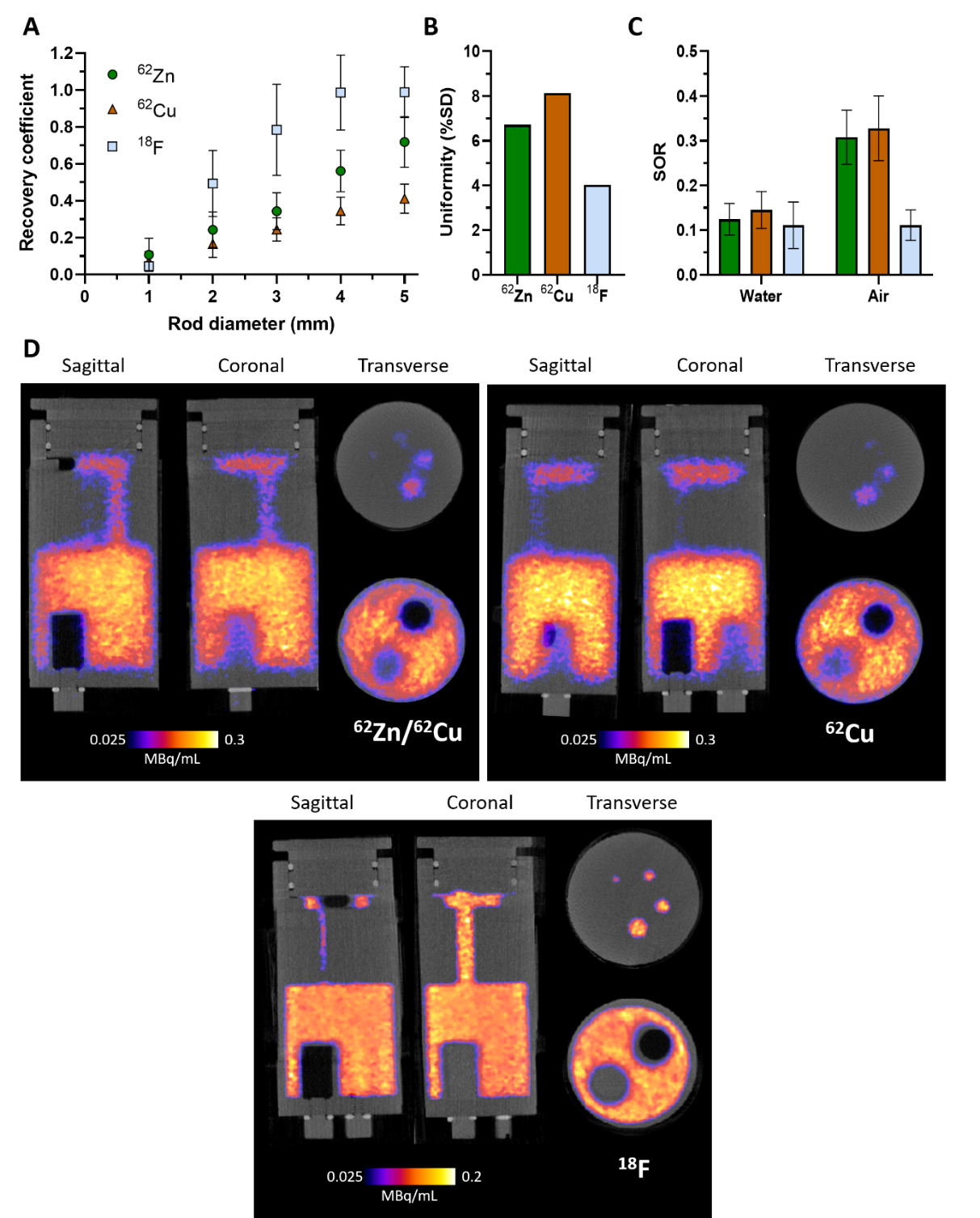


**Fig. S6 Evaluation of image quality of various radionuclides using a NEMA NU-4 phantom.** IQ phantoms were filled with ~4-8 MBq of radioactivity and imaged over 20 mins. Following reconstruction, NEMA NU-4 tests were performed. The RC values of 1-5 mm rods (A), uniformity values (B) and SORs (C) of phantoms imaged were acquired and compared. (D). Representative sagittal, coronal and transverse PET images of 0-20 min summed activity illustrate distinct differences in image quality, notably the poor spatial resolution of ^62^Zn and ^62^Cu compared to ^18^F.

**Fig. S7 Comparison of *ex vivo* biodistribution of [^62^Zn]Zn-citrate (top) and [^64^Cu]Cu-citrate (bottom) in female BALB/c mice (*n* = 4) at 1 h and 24 h after intravenous administration.** Graphs represent mean ± SD. Comparisons were analysed for significance using an unpaired t-test, *, *p* < 0.05; **, *p* < 0.01; ***, *p* < 0.001; ****, *p* < 0.0001.

**Fig. S8 Comparison of *ex vivo* biodistribution of [^62^Zn]Zn-citrate (top) and [^64^Cu]Cu-citrate (bottom) in male BALB/c mice (*n* = 4) at 1 h and 24 h after intravenous administration.** Graphs represent mean ± SD. Comparisons were analysed for significance using an unpaired t-test, *, *p* < 0.05; **, *p* < 0.01; ***, *p* < 0.001.

**Fig. S9 Comparison of *ex vivo* biodistribution of [^62^Zn]Zn-citrate and [^64^Cu]Cu-citrate in male BALB/c mice (*n* = 4) at 1 h and 24 h after intravenous administration.** Female comparisons can be found in Fig. 3 of the main text. Graphs represent mean ± SD. Comparisons were analysed for significance using an unpaired t-test, *, *p* < 0.05; **, *p* < 0.01; ***, *p* < 0.001; ****, *p* < 0.0001.

**Fig. S10 Comparison of *ex vivo* biodistribution of [^62^Zn]Zn-citrate in male and female BALB/c mice (*n* = 4) at 1 h and 24 h after intravenous administration.** Graphs represent mean ± SD. Comparisons were analysed for significance using an unpaired t-test, *, *p* < 0.05; **, *p* < 0.01; ***, *p* < 0.001; ****, *p* < 0.0001.

**Fig. S11 Comparison of *ex vivo* biodistribution of [^64^Cu]Cu-citrate in male and female BALB/c mice (*n* = 4) at 1 h and 24 h after intravenous administration.** Graphs represent mean ± SD. Comparisons were analysed for significance using an unpaired t-test, *, *p* < 0.05; **, *p* < 0.01; ***, *p* < 0.001; ****, *p* < 0.0001.

**Fig. S12 Comparison of *ex vivo* biodistribution of [^62^Zn]Zn-citrate in female BALB/c mice (*n* = 4 per group) fed ad libitum and fasted overnight for 12-16 h at 75 min after intravenous administration.** Graphs represent mean ± SD. Comparisons were analysed for significance using an unpaired t-test, *, *p* < 0.05.
